# Discrimination in healthcare as a barrier to care: experiences of socially disadvantaged populations in France from a nationally representative survey

**DOI:** 10.1186/s12889-019-8124-z

**Published:** 2020-01-09

**Authors:** Joshua G. Rivenbark, Mathieu Ichou

**Affiliations:** 1 Sanford School of Public Policy, Duke University, 201 Science Drive, Durham, NC 27701 USA; 2 Duke University School of Medicine, Duke University, Durham, USA; 3 French Institute for Demographic Studies (INED), Paris, France

**Keywords:** Access to care, Discrimination, International health, Quality of care, Social inequality

## Abstract

**Background:**

People in socially disadvantaged groups face a myriad of challenges to their health. Discrimination, based on group status such as gender, immigration generation, race/ethnicity, or religion, are a well-documented health challenge. However, less is known about experiences of discrimination specifically within healthcare settings, and how it may act as a barrier to healthcare.

**Methods:**

Using data from a nationally representative survey of France (*N* = 21,761) with an oversample of immigrants, we examine rates of reported discrimination in healthcare settings, rates of foregoing healthcare, and whether discrimination could explain disparities in foregoing care across social groups.

**Results:**

Rates of both reporting discrimination within healthcare and reporting foregone care in the past 12 months were generally highest among women, immigrants from Africa or Overseas France, and Muslims. For all of these groups, experiences of discrimination potentially explained significant proportions of their disparity in foregone care (Percent disparity in foregone care explained for: women = 17%, second-generation immigrants = 8%, Overseas France = 13%, North Africa = 22%, Sub-Saharan Africa = 32%, Muslims = 26%). Rates of foregone care were also higher for those of mixed origin and people who reported “Other Religion”, but foregone healthcare was not associated with discrimination for those groups.

**Conclusions:**

Experiences of discrimination within the healthcare setting may present a barrier to healthcare for people that are socially disadvantaged due to gender, immigration, race/ethnicity, or religion. Researchers and policymakers should consider barriers to healthcare that lie within the healthcare experience itself as potential intervention targets.

## Background

People within minority or otherwise socially disadvantaged groups are confronted with a multilevel web of challenges that negatively impact their health and wellbeing [1–3]. Among these numerous factors, research has increasingly focused on experiences of discrimination and how they may relate to individuals’ health [4, 5]. In addition to a direct influence on health via physiologic stress pathways, experiences of discrimination are also thought to influence health indirectly via behavioral responses [6, 7]. Indeed, a meta-analysis reported a significant association between perceptions of discrimination and health-related behaviors such as diet, exercise, sleep, or substance use [8]. However, one health-related behavior that has received comparatively less attention in its association with discrimination is the utilization of healthcare.

Individuals who have experienced discrimination in the past may be more reluctant to seek health care, as they may perceive it as a setting of increased risk for discrimination (i.e., refusal of service or lower quality of care). This may be especially true for those who have experienced discrimination within the health care setting itself. Prior work has hypothesized that experiences of discrimination within the healthcare setting may have a negative effect on individuals’ trust in and satisfaction with the healthcare system, increasing the likelihood of delaying or foregoing seeking care [9–12]. Further, individuals who interact with the healthcare system most often may, simply by greater exposure to the setting, be more likely to experience discrimination in healthcare, and consequently delay or forego future care [13].

Research from the United States (USA) has documented disparities in rates of discrimination in healthcare settings across race/ethnicity, immigrant status, language proficiency, and insurance status [14, 15]. Further research has investigated possible links between discrimination within healthcare and utilization, with mixed findings [9–13, 16]. A large-scale survey conducted in New Zealand documented an association between experiences of racial discrimination within healthcare and lower rates of preventive care use [12], whereas a separate large-scale survey in the USA found that nearly all significant unadjusted associations between discrimination and preventive services were no longer significant once sociodemographic characteristics were controlled for [16]. A number of studies have documented an association between experiences of discrimination within the healthcare setting and delayed or foregone care, both in the USA [9, 10, 17] and in Europe [18]. However, a nationally representative sample of USA women found that discrimination was linked with more frequent healthcare visits, though the authors note that this may not relate to foregone or delayed care [13]. Parallel evidence comes from research among people living with human immunodeficiency virus (HIV), which has consistently shown that higher perceptions of HIV-related discrimination and stigma within care settings is associated with lower retention in care [19, 20].

In addition to the mixed findings above, the existing literature is limited by studies often focusing on a single dimension of social stratification (e.g., disparities in discrimination by race or gender). Research with large-scale nationallyrepresentative samples remains relatively rare [10, 12], making the generalizability of findings to a population level more difficult. Further, the USA remains the site of most existing research on discrimination within healthcare and healthcare utilization, with a small number of studies outside the USA [12, 18]. Finally, although some prior research has tackled the issue of statistical association between discrimination in healthcare settings and healthcare utilization, we know of only one study [16] (and none outside of the USA) that investigates the extent to which discrimination in healthcare can account for gaps in foregone care between groups.

France has a number of distinguishing characteristics that make it an important place for the study of discrimination in healthcare settings and its consequences. France has long been a country of immigration, as significant immigration flows began well before the Second World War [21], and the immigrant population in contemporary France is both numerous and diverse. Among all European countries, France has the second largest population of immigrants born outside the European Union (EU) after Germany, reaching 6 million in 2017 (approximately 9% of the total population) [22]. The largest immigrant groups come from North Africa (Algeria, Morocco, and Tunisia), Southern Europe (especially Portugal), Sub-Saharan Africa, Turkey, Southeast Asia (Vietnam, Cambodia, and Laos) and, more recently, China [23].

France also has a distinct political model of immigrant assimilation and ethnic diversity management, known as the French republican model [24]. Ethnic and racial distinctions are not recognized by the state; as a result ethnic statistics are not collected for official purposes, and ethnic minorities are not considered as targets of social policies [25]. Data and knowledge of discrimination on the basis of ethnicity or migration status are thus extremely scarce, despite the potential insight they could provide on the lived experience of minority groups in France.

Finally, the French healthcare system provides high levels of quality and access to care [26]. It is largely funded by public spending; more than three quarters of total health expenditures are publicly financed. Health insurance has a compulsory and universal coverage [27], and it includes state-funded health services for undocumented immigrants residing in France. This national context, in which the entire population should have access to healthcare, offers a valuable setting for analyzing foregone care and its potential explanatory factors.

In this study, we use data from a nationally representative study in France – with an oversampling of immigrant households – to examine social disparities in discrimination within healthcare, foregone healthcare, and how they are related. These data are of particular interest both for their large-scale, representative nature, and for the demographic diversity of the sample. We leverage these sample strengths and build on prior research by documenting population disparities, both in terms of discrimination and foregone care, across numerous demographic characteristics, including gender, immigrant status, country of origin, and religion. We also explicitly examine the extent to which discrimination in healthcare settings could explain any disparities in foregone healthcare between groups.

## Methods

### Sample

Data come from the Trajectories and Origins (TeO) study [23], a large-scale, nationally representative cross-sectional survey of France. The survey was conducted from 2008 to 2009 with in-person home interviews across France. The sample consisted of 21,761 individuals aged 18 to 59, with oversamples of immigrants and individuals born to at least one immigrant (> 8000 of each group).

### Theoretical framework

Models were conceptualized in line with the adapted Behavioral Model for Vulnerable Populations described by Gelberg and colleagues [28], in which the use of healthcare services represents a health behavior that is influenced by upstream population characteristics. The main population characteristics of interest in this study include demographic characteristics (“predisposing” factors) of gender, ethnicity, immigrant generation, and religion. Other factors that we attempt to account for given the available data include the “predisposing” factors of age, marital status, education, and employment; the “enabling” factor of family income; and the “need” factor of perceived and evaluated health status.

### Measures

#### Healthcare experiences

Discrimination in healthcare was measured with a single yes/no question: “Has a doctor or other medical care worker ever treated you less well or received you less well than other patients?” Likewise, foregone healthcare was also assessed with a yes/no question: “During the past 12 months, have you foregone health care for yourself?”. Each measure was coded dichotomously.

#### Demographic characteristics

As this study was explicitly interested in group disparities in healthcare experiences, we conducted analyses across a series of demographic measures, all of which were self-reported in the survey. Characteristics of interest include gender, immigrant generation (“French-born”, which refers to French-born individuals to French-born parents; first generation immigrant; or second generation immigrant), country of origin (for either the individual or parent, depending on the relevant immigrant generation, grouped into geographic categories), and religion.

#### Covariates

Additional survey items were included as control variables in this study, including age (weighted *M* = 39.1, *SD* = 12.4), marital status (married = 46.7%, weighted) socioeconomic status, and health status. Socioeconomic status was measured with three variables for self-reported monthly income (weighted *M* = 1681€, *SD* = 954€), educational attainment (weighted: less than middle school equivalent = 11.3%, middle school equivalent = 13.3%, vocational training = 26.9%, high school equivalent or higher = 48.6%), and employment status (weighted: employed = 73.1%, unemployed = 8.8%, student = 5.4%, inactive = 12.7%). Health status was also measured with three variables, consisting of self-rated health (weighted *M* = 1.83, *SD* = .79), history of chronic illnesses (yes = 27.1%, weighted), and number of healthcare visits in the last year (weighted: none = 8.2%, once = 24.4%, several = 67.5%).

### Analyses

Analyses proceeded in three main steps. First, we described rates of discrimination in healthcare settings experienced by various groups as the predicted probabilities of experiencing discrimination based on demographic characteristics. We calculated these predicted probabilities from logistic regression models of healthcare discrimination, and we contrasted coefficient estimates against a reference group for statistical comparison. For each demographic factor of interest (gender, migrant generation, origin, and religion), we constructed three nested models. The first model included the demographic predictor, with age and gender (if gender was not the factor investigated) as covariates; the second model added covariates for socioeconomic status; the third model added covariates for health status.

Second, we reported the predicted probabilities of foregoing healthcare across the demographic groups of interest, and then calculated the average marginal effects (AMEs) of the demographic characteristics of interest on those predicted probabilities. We did this by modeling reports of foregone healthcare across three nested logistic regression models: the first included only the demographic factor of interest; the second added discrimination; and the third added all other demographic characteristics, socioeconomic status, and health status. We present our findings as AMEs for two main reasons. First, AMEs are less affected by bias arising from unobserved heterogeneity across nested logistic models than odds ratios or raw logistic regression coefficients [29–31]. Second, we believe that AMEs provide a more intuitive description of effect size than odds ratios or logistic regression coefficients, as AMEs can be read as percentage-point increases in predicted probability.

Finally, we determined how much of the disparities in foregoing healthcare across various groups is potentially explained by experiences of discrimination in healthcare. We did this by calculating the percentage of the Model 1 AME (that is, the AME of a group demographic characteristic) explained by the addition of discrimination as a covariate in Model 2, so that: *% explained* = 1 – (*AME*_*Model 2*_ / *AME*_*Model 1*_). Statistical significance of the “percent explained” was tested by contrasting a demographic characteristic’s AME in Model 2 against the same AME in Model 1. Put another way, we tested the null hypothesis that the addition of discrimination in the model resulted in no change in the estimated AME for a demographic characteristic.

## Results

Descriptive statistics of the sample are shown in Table 1. Overall, the survey-weighted prevalence of reporting discrimination in healthcare settings was 3.9%, with a range of 2.6 to 9.3% across the various demographic groups examined. In bivariate comparisons, significantly higher rates of discrimination were observed for: women compared to men; 1st generation immigrants compared to French-born; those with origins in Overseas France, Africa, and Turkey compared to those from Mainland France; and Muslims and those with no religion compared to Christians.

**Table 1:** Descriptive statistics of study sample and weighted population estimates

Variable	Sample *n*	%^a^	Healthcare Discrimination		Foregone Healthcare	
			%^a^	*p*	%^a^	*p*
Men	10,281	49.2%	3.0%	*ref*	9.9%	*ref*
Women	11,480	50.8%	4.7%	.004	11.9%	.036
French-born	3781	77.7%	3.6%	*ref*	10.4%	*ref*
2nd Generation	8812	11.1%	4.6%	.055	14.2%	<.001
1st Generation	9168	11.2%	4.8%	.013	11.1%	.287
Mainland France	3781	77.7%	3.6%	*ref*	10.4%	*ref*
Overseas France	1345	1.5%	5.9%	.005	15.2%	<.001
North Africa	3706	5.4%	6.4%	<.001	14.2%	<.001
Sub-Saharan Africa	2224	1.8%	7.1%	<.001	12.4%	.072
Turkey	1242	0.8%	6.8%	<.001	10.6%	.893
Southeast Asia	1101	0.5%	4.2%	.535	7.7%	.028
Other Asia	558	1.0%	3.0%	.440	8.5%	.228
Americas	282	0.4%	5.7%	.182	8.9%	.517
Southern Europe	2483	3.4%	2.6%	.080	12.4%	.203
Other Europe	1129	1.6%	3.4%	.750	10.3%	.939
Mixed (1 from FR)	3521	5.5%	3.5%	.857	12.9%	.033
Mixed (no FR)	389	0.4%	4.7%	.353	18.9%	.111
Christian	8405	49.1%	2.9%	*ref*	9.9%	*ref*
No religion	6291	41.2%	4.5%	.009	11.5%	.119
Muslim	5706	7.0%	6.7%	.003	13.5%	.060
Jewish	167	0.5%	2.4%	.234	9.1%	.529
Buddhist	579	0.6%	9.3%	.322	6.2%	.065
Hindu/Sikh	68	0.1%	3.8%	.758	13.2%	.677
Other Religion	203	0.6%	6.2%	.547	22.0%	.065
Refuse/Unsure	318	1.1%	2.6%	.201	13.1%	.746
Total	21,761	100.0%	3.9%	–	10.9%	–

^a^These estimates are population-weighted

**Fig. 1:**
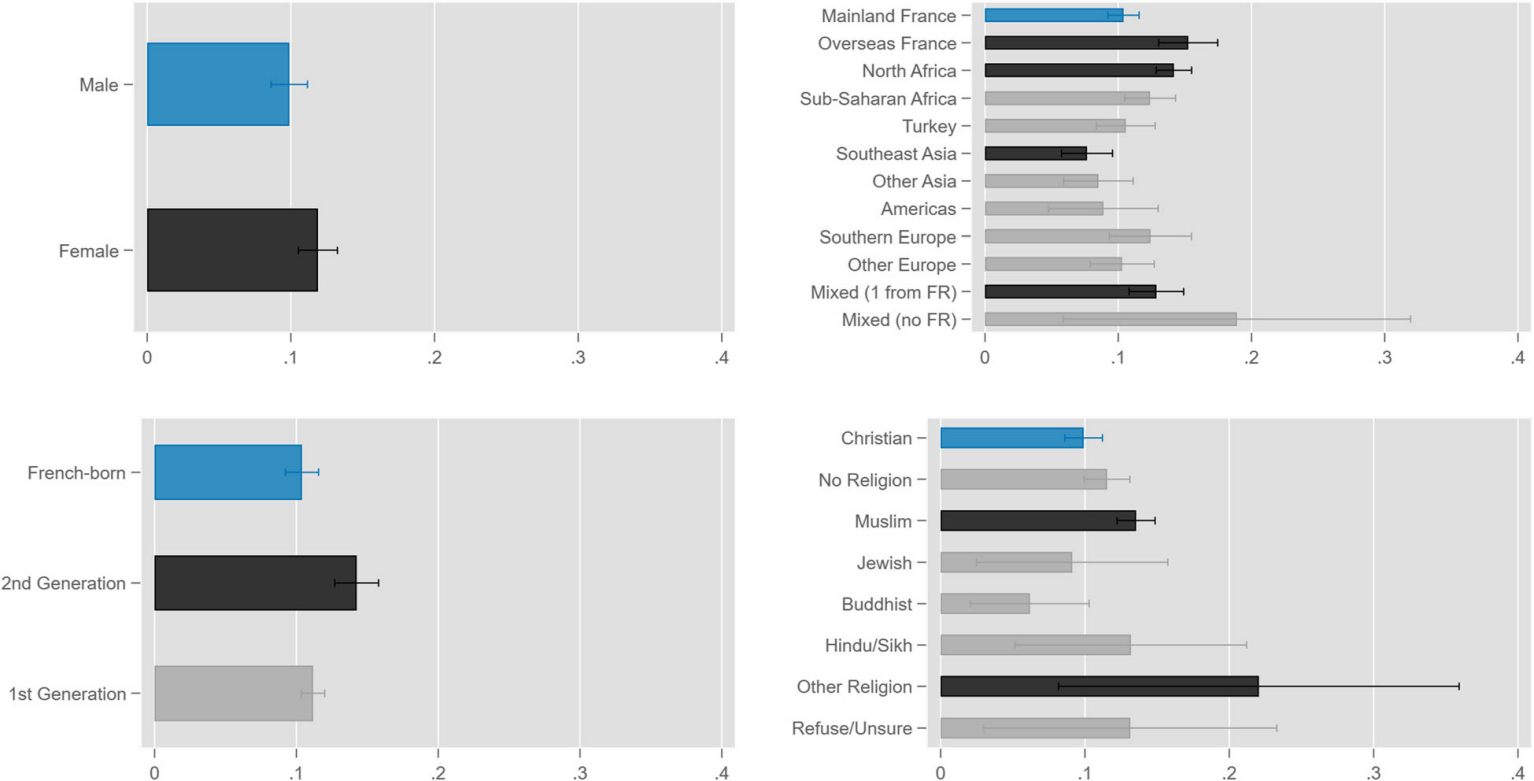
Predicted probabilities of foregoing healthcare. Predicted probabilities were derived from logistic regression of foregoing healthcare on demographic characteristics, with no covariates (*N =* 21,729). Bar colors represent statistical significance in logistic regression of foregoing healthcare on demographic characteristics: blue = reference group; black = (*p* < .05); grey = (*p* > .05)

Also seen in Table 1, the survey-weighted rate of foregone healthcare was 10.9% overall, ranging from 6.2 to 22.0% across demographic groups. Bivariate comparison tests are displayed in the table, and represented graphically in Fig. 1, as predicted probabilities of foregoing healthcare across demographic groups. Blue bars correspond to the reference groups, black bars indicate significant difference from reference group levels, and grey bars indicate no significant difference. The probability of foregoing care was higher for: women compared to men; second-generation immigrants compared to French-born; people with origins in Overseas France, North Africa, or mixed origin (partially from France) compared to those from Mainland France; and Muslims and those who reported “Other Religion” compared to Christians. In contrast, the probability of foregoing care was lower for people of Southeast Asian origin.

Predicted probabilities of foregoing healthcare were then calculated across a series of nested models; the results are displayed in Table 2 and illustrate three main findings. First, discrimination in healthcare settings was strongly associated with having foregone healthcare across all models in which it was included (Models 2 and 3). In the fully adjusted Model 3, the AME of discrimination was 0.14 – the largest effect size of all covariates, corresponding to a 14-percentage point increase in the predicted probability of foregoing care. Second, the AMEs associated with women, Muslim, Buddhist, or other religion, as well as origin in North Africa or Southeast Asia, which were statistically significant in Models 1 and 2, were no longer significant with the addition of other sociodemographic factors as covariates in Model 3. Third, the AME of certain demographic characteristics was not fully explained by any of the added covariates (i.e., it remained statistically significant even in the most strictly controlled model). Namely, in Model 3 there were significant AMEs of foregoing healthcare for second-generation immigrants, those with an origin in Overseas France, or those with mixed origin (regardless of whether or not it was partially from France).

**Table 2:** Average marginal effects (AMEs) of demographic characteristics and reports of discrimination for predicting foregoing healthcare

	*Model 1*		*Model 2*		*Model 3*	
	*AME*	*s.e*	*AME*	*s.e*	*AME*	*s.e*
Men (ref)	–	–	–	–	–	–
Women	0.023**	0.010	0.019*	0.010	0.011	0.010
No HC discrim (ref)			–	–	–	–
HC discrim			0.222***	0.039	0.140***	0.033
*F*-statistic	–		*F* = 59.7***		*F* = 5.59***	
*N*	19,202		19,202		19,202	
Mainland France (ref)	–	–	–	–	–	–
Overseas France	0.040***	0.015	0.035**	0.014	0.023*	0.013
North Africa	0.036***	0.01	0.028***	0.01	0.014	0.015
Sub-Saharan Africa	0.025*	0.013	0.017	0.012	0.007	0.013
Turkey	−0.004	0.014	−0.011	0.013	−0.018	0.016
Southeast Asia	− 0.029**	0.012	− 0.030**	0.012	− 0.022	0.017
Other Asia	−0.018	0.016	−0.016	0.016	−0.005	0.02
Americas	−0.010	0.024	−0.015	0.022	−0.001	0.025
Southern Europe	0.018	0.018	0.021	0.018	0.023	0.019
Other Europe	−0.004	0.015	−0.003	0.015	0.005	0.016
Mixed (1 from FR)	0.027*	0.014	0.027*	0.014	0.026*	0.014
Mixed (no FR)	0.044**	0.022	0.044**	0.022	0.046**	0.022
No HC discrim (ref)			–	–	–	–
HC discrim			0.224***	0.04	0.140***	0.033
*F*-statistic	–		*F* = 59.8***		*F* = 6.75***	
*N*	19,202		19,202		19,202	
French-born (ref)	–	–	–	–	–	–
2nd Generation	0.039***	0.011	0.036***	0.011	0.032***	0.011
1st Generation	0.007	0.008	0.005	0.008	0.005	0.009
No HC discrim (ref)			–	–	–	–
HC discrim			0.225***	0.04	0.139***	0.033
*F*-statistic	–		*F* = 60.3***		*F* = 6.85***	
*N*	19,202		19,202		19,202	
Christian (ref)	–	–	–	–	–	–
No Religion	0.017	0.011	0.013	0.011	0.010	0.011
Muslim	0.034***	0.01	0.025**	0.01	0.010	0.016
Jewish	−0.041*	0.024	−0.040	0.025	−0.044*	0.026
Buddhist	−0.040	0.025	−0.039	0.025	−0.025	0.033
Hindu/Sikh	0.034	0.046	0.031	0.046	0.013	0.045
Other Religion	0.134*	0.079	0.122	0.075	0.085	0.067
Refuse/NSP	0.081	0.071	0.084	0.072	0.057	0.064
No HC discrim (ref)			–	–	–	–
HC discrim			0.221***	0.039	0.140***	0.033
*F*-statistic	–		*F* = 58.5***		*F* = 6.33***	
*N*	19,202		19,202		19,202	

Each panel (i.e., gender, origin, migrant generation, religion) is a separate set of nested logistic regression models predicting foregoing healthcare. Model 1 contains only the demographic characteristic of interest as a predictor. Model 2 adds discrimination in healthcare as a predictor, Model 3 then adds other covariates, including demographic characteristics, measures of socioeconomic status, and measures of health status. For conciseness, only the average marginal effects of demographic characteristics of interest and reported discrimination in healthcare are tabulated. HC: healthcare. *:*p* < .1; **:*p* < .05; ***:*p* < .01

Finally, we examined the proportion of the disparities in foregone healthcare potentially explained by reporting discrimination in healthcare settings; the results are shown in Table 3. Discrimination explained a statistically significant proportion of the disparity for women relative to men (17%), second-generation immigrants relative to French-born individuals (8%), people with origins in Overseas France (13%), North Africa (22%), and Sub-Saharan Africa (32%) relative to those with origins in Mainland France, and Muslims (26%) relative to Christians.

**Table 3:** Proportion of disparities in foregoing healthcare explained by discrimination in healthcare

Variable	Proportion of disparity explained	*p*
Men	*(ref)*	*(ref)*
Women	0.17	0.014
French-born	*(ref)*	*(ref)*
2nd Generation	0.08	0.053
Mainland France	*(ref)*	*(ref)*
Overseas France	0.13	0.049
North Africa	0.22	< 0.001
Sub-Saharan Africa	0.32	0.003
Southeast Asia	−0.03	0.705
Mixed (no FR)	0.00	0.880
Christian	*(ref)*	*(ref)*
Muslim	0.26	< 0.001
Buddhist	−0.03	0.312
Other Religion	0.09	0.307

The proportion explained is calculated from coefficients in Table 2, as (1 – (*Model 1 AME / Model 2 AME*)). The *p* value refers to statistically contrasting the AME in Model 1 and Model 2; that is, it represents a test of the null hypothesis that the proportion explained is equal to zero. Only those variables with an observed AME in Model 1 are tabulated here, as they represent baseline gaps in foregoing healthcare across demographic characteristics

## Discussion

This study used data from a national population-representative survey to look at the experiences of people who are socially disadvantaged due to gender, immigration, race/ethnicity, and religion, within the healthcare setting in France. We examined rates of reported discrimination and how they may explain disparities in rates of foregoing healthcare among those groups. Overall, our findings suggest that discrimination in healthcare is associated with foregoing medical care, and that this is especially important for women and people in minority racial or religious groups.

More specifically, our results suggest three main points. First, we showed that disadvantaged social groups – particularly women, immigrants, those of African origin, and Muslim religion – are more likely to have experienced discrimination in healthcare settings. The population prevalence of discrimination of 3.9%, which was in line with prior research across more than 30 European countries documenting national rates of discrimination in primary care between 1.4 and 12.8% [32], obscures the heterogeneity across groups, with rates nearly doubling for disadvantaged groups. For many of these groups, this finding is consistent with a broad base of existing literature, as they have been shown to face higher risks of discrimination in French society. Immigrants and their children from Sub-Saharan Africa, North Africa, and the French overseas territories report higher rates of perceived discrimination, measured through both general and setting-specific discrimination questions (at school, on the labor or housing markets, etc.) [33]. These minority groups also face racism more frequently [34]. Among religious groups, our observation of a high rate of discrimination against Muslims in the healthcare system echoes previous findings of discrimination in other settings [33], especially the labor market [35], and high levels of anti-Muslim prejudice in French society overall [36]. In contrast, there seems to be a specificity of the healthcare setting for women. Our findings are consistent with qualitative evidence showing that women tend to report discrimination in healthcare settings more often than men [37], but differ from findings in other settings (school, the labor and housing markets) where women are less likely to perceive discrimination [33]. One possible factor contributing toward this setting-specificity could be the higher rate of healthcare utilization by women, which would in turn increase their exposure to the possibility of experiencing discrimination within that setting.

Second, our analysis documented disparities in the rates of foregoing medical care across populations of social disadvantage due to gender, immigration, race/ethnicity, and religion. Many of the groups with higher rates of foregoing healthcare were the same as those who reported higher rates of discrimination in healthcare – women, immigrants (though second-generation, rather than first), people with origins in Africa or Overseas France, and Muslims. Other groups with comparatively high rates of foregoing healthcare were those with mixed origins, and those who reported as “Other Religion”. For some groups, these findings are in line previous research on foregoing care: for example, there is evidence of higher rates of foregoing healthcare among adult women in Sweden and adolescent girls in the USA [18, 38]. Similarly, prior research has consistently documented higher rates of foregoing care among disadvantaged racial and ethnic minority groups in the US [39, 40]. However, there is less existing research on migrant generation and foregoing care, and our finding of higher rates of foregoing care among second-generation immigrants in France differs from a study of immigrant children in the USA, which documented higher rates of foregone care for first-generation immigrants, but not second-generation [41]. We are not aware of other reports of foregone healthcare by religion.

Finally, we examined the potential explanatory role of experiences of discrimination in the healthcare setting on foregoing healthcare. We found reports of discrimination to be robustly linked with foregoing care: in our fully adjusted model of foregoing care, discrimination in the healthcare setting was associated with an average 14 percentage-point increase in the predicted probability of foregoing care. Of note, this contrasts with a prior study that found the link between discrimination and decreased healthcare utilization to be explained by socioeconomic status [16]. These findings can also be considered alongside a USA-based study that found discrimination to be associated with more frequent healthcare visits [13] together, these studies are consistent with the model described in this paper, in which healthcare need (observed as frequency of visits) is an enabling factor for discrimination in healthcare, which results in a higher likelihood of foregoing future care [28]. Overall, findings in this study are consistent with existing research on discrimination as a barrier to healthcare: in addition to the previously mentioned Swedish study linking discrimination with foregone healthcare, qualitative research from Spain has described experiences of discrimination as a factor limiting access to healthcare [42], and experiences of discrimination have been linked to avoiding dental care in Australia [43].

We also contextualized this relationship by determining the potential proportion of disparities in foregoing care that could be explained by experiences of discrimination in healthcare. Groups for whom discrimination explained an especially large proportion of disparities in foregone care were people with origins in Sub-Saharan Africa (32%) and Muslims (26%). Also of note were women (17%); although the proportion explained was lower for women than for some other groups, the fact that they constitute half of the population points toward a large potential effect of discrimination when considered at the level of French society. Interestingly, the proportion of the disparity in foregoing care for second-generation immigrants explained by discrimination was small (8%). Taken together with the findings by region of origin, this suggests that discrimination may be of particular importance for healthcare utilization among immigrants who are more readily racialized based on their appearance and face higher levels of racism already.

This study has a number of limitations that should be noted. First, this was cross-sectional and thus no causal inference regarding discrimination and foregoing healthcare can be made – it is for this reason that results are framed in terms of the potential explanatory nature of discrimination. Future studies should consider possible natural experiments or other quasi-experimental designs in order to more rigorously test any causal relation between discrimination and foregoing healthcare. Second, we used a single-item measure of discrimination in healthcare settings, framed as being treated poorly compared to other patients. It is possible that a different assessment of discrimination, such as an adapted version of the Everyday Discrimination Scale [44], would reveal a different pattern of rates of discrimination. Third, we did not examine the specific type of healthcare that individuals reported having foregone, and thus do not know to what extent the foregone care was necessary. Finally, although this study was nationally representative of France, findings may be dependent on the societal dynamics and healthcare setting specific to France at that time (2008–2009), and consequently not generalizable to other settings. However, the rates of both discrimination in healthcare settings and of foregoing care are generally similar to those described in Sweden [18] – which has a different healthcare system and a more homogenous population – suggesting that similar trends may exist at least in other parts of Europe. Further, given the contemporary increase in far-right voting and associated anti-immigration politics in France, we would hypothesize that our estimates here represent lower bounds for experiences of discrimination in the present.

With these potential limitations in mind, the implications of this study can be discussed. We observe disparities between social groups in terms of discrimination in healthcare settings – a negative phenomenon itself – as well rates of foregone healthcare, an important hurdle in the functioning of any health system [45]. The affected groups represent large sections of French society (e.g., women, major immigrant groups, etc.), suggesting a substantial burden when considered at the national level. These disparities stand in opposition to the global goals of health equity [46–48], and should be considered in the discussion and design of interventions and health policies. Suggested interventions to reduce discrimination in healthcare settings include provider-level interventions, grounded in psychology research, that aim to improve provider understanding of bias and increase perspective-taking and empathetic behaviors [49], such as an intervention involving feedback on biased behaviors and interactions with a virtual patient that may reduce racial bias in pain medicine prescribing [50]. More systemic actions include policies that increase organizational accountability for discrimination, or social marketing campaigns that aim to shift population norms with anti-discrimination messaging [51]. The robust linkage between experiences of discrimination and foregoing healthcare observed in this study, especially among women, immigrants of African origin, and Muslims, adds additional context to the web of barriers that people in socially disadvantaged groups face and points to potential high-priority groups around which interventions may be structured.

## Conclusion

The health status of disadvantaged and minority populations is a topic of increasing policy and scientific relevance for many countries around the world [52–54]. This study provides evidence that discrimination within healthcare settings may partially explain disparities in rates of foregone healthcare, contributing to the health inequalities observed across various disadvantaged groups. Researchers and policymakers who aim to improve the health of disadvantaged groups should be mindful that some barriers to healthcare for disadvantaged populations may lie in the experiences of healthcare itself, and those experiences are a potential place of action from which future policy and research can proceed.

## Data Availability

The data that support the findings of this study are available from the French Institut national d’études démographiques (INED), but restrictions apply to the availability of these data, which were used under license for the current study, and so are not publicly available. Data are however available from the authors upon reasonable request and with permission of INED.

## Abbreviations

AME: Average marginal effects
EU: European Union
HIV: Human immunodeficiency virus
TeO: Trajectories and Origins
USA: United States of America
